# Digital templating cementless short stem total hip arthroplasty: is there a difference in planning adherence between the direct anterior approach and minimally invasive anterolateral approach?

**DOI:** 10.1007/s00402-022-04374-9

**Published:** 2022-02-18

**Authors:** Matthias Luger, Sandra Feldler, Bernhard Schauer, Rainer Hochgatterer, Tobias Gotterbarm, Antonio Klasan

**Affiliations:** 1 Department for Orthopaedics and Traumatology, Kepler University Hospital GmbH, Krankenhausstrasse 9, 4020 Linz, Austria; 2 Johannes Kepler University Linz, Altenberger Strasse 69, 4040 Linz, Austria

**Keywords:** Digital templating, Short stem, Fitmore, Total hip arthroplasty, Minimally invasive, Anterolateral approach, Direct anterior approach

## Abstract

**Purpose:**

Minimally invasive approaches (MIS) in total hip arthroplasty (THA) show inconsistent findings regarding planning adherence in digital templating. The purpose of this study is to evaluate any difference in planning adherence between the direct anterior approach (DAA) and an anterolateral MIS approach (AL MIS) in cementless short stem THA.

**Methods:**

A single surgeon series of 222 THAs in 208 patients with an uncemented short curved stem and a bi-hemispherical acetabular cup were screened for inclusion. A total of 118 THAs were implanted via the DAA and 72 THAs via the AL MIS were included. The planning adherence for the offset option, stem size and the acetabular cup were retrospectively evaluated.

**Results:**

Planning adherence for cup size (perfect match: *p* = 0.763; ± 1 size: *p* = 0.124), offset option (0.125) and stem size (perfect match:* p* = 0.275; ± 1 size: *p* = 0.552) did not show any statistical significance. Preoperative diagnosis of avascular necrosis of the femoral head in AL MIS approach (OR 6.045; CI 1.153–31.696) or mild hip dysplasia in the general cohort poses (OR 11.789; CI 1.386–100.293) a significant risk for inadequate prediction of the offset option. Conclusion: digital templating for THA with an uncemented short curved stem and a bi-hemispherical acetabular cup show comparable results between a direct anterior approach and a minimally invasive anterolateral approach in supine position. Surgeons should be aware of a low planning adherence for this type of short stem in minimally invasive approaches.

**Conclusion:**

Digital templating for THA with an uncemented short curved stem and a bi-hemispherical acetabular cup show comparable results between a direct anterior approach and a minimally invasive anterolateral approach in supine position. Surgeons should be aware of a low planning adherence for this type of short stem in minimally invasive approaches.

## Introduction

Preoperative digital templating in total hip arthroplasty (THA) is an integral part in selecting the correct implant size [1, 2]. An underestimation of component size can lead to instability and component loosening, an overestimation of component size can lead to periprosthetic fractures of both, the acetabular and femoral components [2–4]. Additionally, preoperative planning is essential for planning the correct restoration of the hip offset and avoiding leg length discrepancy [1, 5]. The accuracy of preoperative digital templating ranges from 78 to 98% within one size for cementless straight stems and from 80 to 90% within one size for the acetabular component [1, 2, 6–9].

Short stems for total hip arthroplasty have gained popularity over the last few years in parallel with minimally invasive surgical approaches (MIS) [1]. Short stems facilitate soft-tissue-sparing minimally invasive approaches. Additionally, cementless short stems are associated with superior bone preservation of the proximal femur and reduced stress shielding [1, 2]. However, the results for planning adherence for cementless short stems shows mixed results [3–6]. Rivera et al. [6] demonstrated an association between the implantation of cementless short stems via a direct anterior approach (DAA) and an increased risk of underestimating the size of the femoral stem compared to posterior approach (PA) in acetate templating. In case of minimally invasive anterolateral approach the same cementless short stem showed poorer results in planning adherence [3]. Additionally, lower canal fill indices were reported in the use of cementless short stems implanted via a DAA or a MIS anterolateral approach [7]. This might be due to reduced femoral exposure with minimally invasive approaches and therefore impaired femoral broaching. However, Shemesh et al. [8] did not find any significant difference in planning accuracy between the DAA and the PA in cementless straight stem THA. Schmidutz et al. reported a comparable planning adherence for short stem THA with 89.0% (± 1 stem size) compared to straight stem THA with 88.5% (± 1 stem size).

Planning adherence in preoperative digital templating could be influenced by the used approach as well as by the used implant. As minimally invasive approaches are considered to be a factor for stem undersizing, we wanted to compare the planning adherence in minimally invasive short stem THA. Therefore, the aim of this study was to analyse whether there is a difference between the direct anterior approach and a minimally invasive anterolateral approach in supine position regarding effective adherence in digital templating for cementless short stems.

## Methods

### Study design

In this retrospective study a consecutive series of 222 THAs (208 patients, mean age 66.9 years ± 11.4, min.: 30.8, max.: 87.5 years) between 2014 and 2019 were screened for inclusion. Prior to inclusion the study was approved by the local ethics committee (EK-No.: 1239/2019). All procedures performed in studies involving human participants were in accordance with the ethical standards of the institutional and/or national research committee and with the 1964 Helsinki declaration and its later amendments or comparable ethical standards. Informed consent was not required because of the retrospective study design.

All THAs were performed using either a DAA or a MIS anterolateral approach in supine position. All included patients were operated for either severe, end-stage osteoarthritis, end-stage avascular necrosis of the femoral head (AVN) or mild hip dysplasia (Crowe I). Exclusion criteria were a history of prior surgery on the affected hip, THA for femoral neck fractures, post-traumatic osteoarthritis, and complex deformities (i.e., severe hip dysplasia (Crowe > 1), Legg-Calve-Perthes) because of possible influence on planning adherence because of different anatomical variances. Periprosthetic fractures led to exclusion of this study because of the possible influence on the component sizing. In case of bilateral one-stage implantation the patients were excluded because of statistical reasons. Patients with bilateral two-staged implantations were included with the first case. Additionally, previous surgery on the contralateral side might influence preoperative templating and choose of implant size for the second implantation.

### Surgical procedure and implants

The procedures were performed by a single fellowship trained surgeon. DAA was carried out in a supine position on a standard operating table as previously described [10, 11]. Minimally invasive anterolateral approach also was carried out on a standard operating table in supine position [12]. Fluoroscopy was not routinely used. The standardized peri- and postoperative protocol was identical in both groups, including single-shot antibiotics (Cefuroxime 1,5 g i.v. perioperatively), weight-bearing as tolerated, Indomethacin 75 mg daily for the prevention of heterotopic ossification for four days and 40 mg low-molecular weight heparin or Rivaroxaban 10 mg for 28 days postoperatively as prophylaxis for deep vein thrombosis.

All patients received an Allofit/-S^®^ acetabular cup and a Fitmore^®^ curved short stem (both Zimmer Inc., Warsaw, IN, USA). The Fitmore^®^ hip stem is an uncemented short curved hip stem available in four different offset options with a CCD-Angle ranging from 140° to 127° [A (140°), B (137°), B extended (129°) and C (127°)]. Every offset option is available in 14 different sizes. Allofit^®^ cup is a bi-hemispherical press fit cup with sizes ranging from 42 to 68 mm.

### Preoperative X‑ray technique

A standardized preoperative digital radiograph anterior–posterior view of the hip was obtained in every case. Radiographs were taken with the patient in standing position and with both legs in 15° internal rotation. The beam was centred on the symphysis pubis. A standardized metallic radiopaque ball with a diameter of 25 mm was used as a reference for determining the magnification factor.

### Digital templating and radiological measurement

Digital templating was carried out with mediCAD^®^ version 5.1 (Hectec GmbH, Altdorf, Germany) in a standardized manner. At first scaling and calculating the right magnification factor was done automatically by the software with the metallic radiopaque ball as the reference. Then the centre of rotation, the proximal femoral shaft axis and the leg length discrepancy were determined. After that the correct size for the acetabular component was determined. The acetabular component was placed at the floor of the acetabulum, as this was the intended final position intraoperatively. Next the size of the femoral component was templated beginning with the correct offset option. The aim in templating a Fitmore curved short hip stem is to restore the anatomical offset by confirming that the medial curve of the stem follows closely the inner medial line of the cortex closely in the calcar region when the stem is in axis with the femoral canal. After choosing the correct stem family, the appropriate stem size is selected. The appropriate stem size is selected by choosing the stem which fills the intramedullary canal entirely. In general, surgeons tend to obey the predetermined offset option. In case of instability in trial reduction, they chose a higher offset option instead of the predetermined offset option.

### Statistical analysis

Statistical analysis was calculated with SPSS version 26 (IBM SPSS statistics, Chicago, IL, USA). The level of significance was *p* < 0.05. Descriptive analysis was done for the parameters age, sex, BMI, and diagnosis. A Shapiro–Wilk test was performed for testing for normality distribution. As not all variables were normally distributed, non-parametric testing was performed. For testing differences in patient demographics between both groups a Fisher’s exact test (laterality; gender), a Mann–Whitney-*U*-Test (age; BMI; diagnosis) were performed. For testing in differences according to BMI-groups a Kruskal–Wallis-Test was performed. A Fisher’s exact test was calculated to test for significant differences in planning adherence between DAA and AL MIS approach. A univariate logistic regression analysis was performed to detect differences between the surgical approaches and to identify possible confounders on effective adherence of digital templating. Analysed factors with possible influence on planning accuracy were surgical approach, gender, age, diagnosis and BMI.

## Results

A total of 190 THAs met the inclusion criteria. Indication for THA was primary osteoarthrosis in 169 patients (88.9%), avascular necrosis in 14 patients (7.4%) and hip arthrosis due to hip dysplasia in 7 patients (3.7%). Patients were then assigned to two groups according to the used approach. Group A consisted of 118 patients operated using direct anterior approach. Group B consisted of 72 patients operated using minimally invasive supine anterolateral approach. Detailed demographic data for both groups are shown in Table 1. There were no baseline differences between both groups, Table 1. Testing for BMI did not show any differences in average BMI (*p* = 0.304) and divided into subgroups (*p* = 0.405) between both approaches, Table 1.

**Table 1:** Patient demographics

	Total	DAA	AL MIS	*P* value
Gender (f:m)	96:94	58:60	38:34	0.656
Age at operation	67 ± 11.4	66.8 ± 11.2	67.2 ± 11.6	0.854
Diagnosis				0.062
Primary OA	169 (88.9%)	109 (92.4%)	60 (83.3%)	
AVN	14 (7.4%)	5 (4.2%)	9 (12.5%)	
Hip dysplasia	7 (3.7%)	4 (3.4%)	3 (4.2%)	
Laterality (l:r)	98:92	59:59	39:33	0.654
BMI at surgery	27.7 ± 4.7	27.4 ± 4.5	28.2 ± 4.9	0.304
BMI ≤ 25 kg/m^2^	61 (32.1%)	38 (32.2%)	23 (31.9%)	0.405
BMI > 25–29.99 kg/m^2^	82 (43.2%)	55 (46.6%)	27 (37.5%)	
BMI 30–34.99 kg/m^2^	35 (18.4%)	19 (16.1%)	16 (22.2%)	
BMI ≥ 35 kg/m^2^	12 (6.3%)	6 (5.1%)	6 (8.3%)	

Gender: *f* female, *m* male, *primary OA* primary osteoarthritis, *AVN* avascular necrosis of the femoral head, laterality: *l* left, *r* right, *BMI* Body Mass Index (kg/m^2^)

### Planning accuracy for offset option

The exact offset option as templated was used in 62.6% in the general cohort, and in 66.4% in DAA and in 55.6% in AL MIS, Table 2. The implantation of one offset option bigger than templated was similar in both groups (DAA: 21.2% vs. AL MIS 27.8%), Table 2. The implantation of an offset option one size smaller than templated was less common in both groups (DAA: 11.0% vs. AL MIS 13.9%), Table 2. Testing for statistically significant difference for the offset option between both approaches could not be found (*p* = 0.125), Table 3.

**Table 2:** Planning adherence for offset option, stem size and cup size

Offset option	Perfect match	− 1 offset option	+ 1 offset option	− 2 offset options	+ 2 offset options
Total	119 (62.6%)	45 (23.7%)	23 (12.1%)	3 (1.6%)	0 (0.0%)
DAA	79 (66.4%)	25 (21.2%)	13 (11.0%)	1 (0.8%)	0 (0.0%)
AL MIS	40 (55.6%)	20 (27.8%)	10 (13.9%)	2 (2.8%)	0 (0.0%)

Stem size	Perfect match	± 1 size	± 2 sizes	± 3 sizes and more	Offset option not correct
Total	41 (21.6%)	49 (25.8%)	23 (12.1%)	6 (3.2%)	71 (37.4%)
DAA	22 (18.6%)	36 (30.5%)	17 (14.4%)	4 (3.4%)	39 (33.1%)
AL MIS	19 (26.4%)	13 (18.1%)	6 (8.3%)	2 (2.8%)	32 (44.4%)

Cup size	Perfect match	± 1 size	± 2 sizes	± 3 sizes and more	
Total	83 (43.7%)	72 (37.9%)	29 (15.3%)	6 (3.2%)	
DAA	53 (44.9%)	39 (33.1%)	21 (17.8%)	5 (4.2%)	
AL MIS	30 (41.7%)	33 (45.8%)	8 (11.1%)	1 (1.4%)	

-1 offset option – offset option one size smaller templated than intraoperatively used +1 offset option – offset option one size bigger templated than intraoperatively used -2 offset options – offset option two sizes smaller templated than intraoperatively used +2 offset options – offset option two size bigger templated than intraoperatively used *DAA* direct anterior approach,* AL MIS* minimally-invasive anterolateral approach

**Table 3:** Fisher’s exact test for differences between DAA and AL MIS

	*P* value
Cup perfect match	0.763
Cup ± 1 size	0.124
Offset Option perfect match	0.125
Stem size perfect match	0.275
Stem Size ± 1 size	0.552

*DAA* direct anterior approach, *AL MIS* minimally invasive anterolateral approach

### Planning accuracy for stem size

The exact offset option and stem size was correctly predicted in 21.6% in the general cohort, Table 2 In the DAA cohort, the stem size as templated was used in 18.6%, Table 2. In the AL MIS cohort, the exact stem size as templated was implanted in 26.4%, Table 2. The use of a stem size within ± 1 size was more common in DAA with 30.5% compared to 18.1% in AL MIS. A stem size ± 2 sizes was used in 12.1% (*n* = 23) in total, Table 2. Similarly, a stem size within ± 2 sizes was chosen more commonly in DAA compared to AL MIS (14.4% vs. 8.3%) with the AL MIS, Table 2. Testing for statistically significant differences for the stem size for a perfect match and for adherence ± 1 size between both approaches could not be found (*p* = 0.275; 0.552), Table 3.

### Planning accuracy for cup size

The exact cup size as preoperatively planned was used in 43.7% (*n* = 83) in the general cohort, Table 2. The cup size as templated was used in 44.9% in the DAA and in 41.7% in the AL MIS approach, Table 2. A cup size within ± 1 size was more common in AL MIS approach (DAA: 33.1% vs. AL MIS: 45.8%), while a cup size within ± 2 sizes was more common in the DAA (DAA: 17.8% vs. AL MIS: 11.1%), Table 2. Testing for statistically significant differences for the cup size for a perfect match and for adherence ± 1 size between both approaches could not be found (*p* = 0.763; 0.124), Table 3.

### Regression analysis

A univariate regression analysis was performed to identify risk factors for reduced planning accuracy. Detailed results for the regression analysis are given in Table 4. The preoperative diagnosis of AVN or mild hip dysplasia was shown to reduce planning accuracy, Table 5. AVN showed a significantly increased OR for the planning adherence of the offset option (OR 6.045; 1.153–31.696). Hip dysplasia showed a significantly increased risk for the planning adherence of the offset option in the general cohort (OR 11.789; CI1.386–100.293), Table 5.

**Table 4:** Univariate logistic regression

Cup	Gender	Age	BMI	Approach
Total	1.417 (0.797–2.521)	1.007 (0.982–1.033)	1.037 (0.974–1.105)	1.142 (0.631–2.065)
*P* value	0.235	0.571	0.256	0.661
DAA	1.139 (0.551–2.354)	0.994 (0.962–1.027)	1.030 (0.948–1.119)	–
* P* value	0.725	0.728	0.492	
AL MIS	2.091 (0.801–5.458)	1.028 (0.986–1.072)	1.046 (0.947–1.156)	–
*P* value	0.132	0.188	0.371	
Offset option				
Total	1.295 (0.719–2.335)	0.995 (0.969–1.021)	1.024 (0.962–1.091)	1.621 (0.887–2.961)
*P* value	0.389	0.697	0.454	0.116
DAA	1.396 (0.645–3.020)	1.001 (0.967–1.036)	1.030 (0.947–1.122)	–
*P* value	0.396	0.972	0.489	
AL MIS	1.222 (0.481–3.103)	0.986 (0.947–1.027)	1.007 (0.916–1.107)	–
*P* value	0.673	0.495	0.885	
Stem Size				
Total	0.915 (0.458–1.827)	1.011 (0.981–1.042)	1.029 (0.952–1.112)	0.609 (0.318–1.287)
*P* value	0.801	0.480	0.474	0.210
DAA	0.833 (0.329–2.111)	1.022 (0.981–1.065)	1.033 (0.926–1.153)	–
*P* value	0.701	0.294	0.562	
AL MIS	0.992 (0.347–2.834)	0.998 (0.954–1.045)	1.035 (0.925–1.157)	–
*P* value	0.982	0.939	0.552	

*BMI* Body Mass Index (kg/m^2^), *DAA* direct anterior approach, *AL* *MIS* minimally invasive anterolateral approach

**Table 5:** Univariate logistic regression for planning adherence stratified by approach and diagnosis

	Cup		Offset option		Stem size	
	OR (CI)	*P* value	OR (CI)	*P* value	OR (CI)	*P* value
Total						
OA	1.000	–	1.000	–	1.000	–
AVN	1.090 (0.362–3.277)	0.879	2.620 (0.867–7.914)	0.119	2.590 (0.782–8.584)	0.119
Hip dysplasia	4.903 (0.578–41.616)	0.145	11.789 (1.386–100.293)	**0.024**	NV	–
DAA						
OA	1.000	–	1.000	–	1.000	–
AVN	1.271 (0.204–7.912)	0.797	0.529 (0.057–4.905)	0.575	1.585 (0.255–9.862)	0.622
Hip dysplasia	2.542 (0.256–25.214)	0.425	6.343 (0.637–63.177)	0.115	NV	-
AL MIS						
OA	1.000	–	1.000	–	1.000	–
AVN	0.956 (0.233–3.917)	0.950	6.045 (1.153–31.696)	**0.033**	1.384 (0.261–7.342)	0.703
Hip dysplasia	NV	–	NV	–	NV	–

*OA* primary osteoarthritis,* AVN* avascular necrosis of the femoral heard,* DAA* direct anterior approach,* AL MIS* minimally invasive anterolateral approach,* OR* Odds ratio,* CI* 95% Confidence Interval

## Discussion

In this study, the planning adherence for a short, curved cementless hip stem and a bi-hemispherical acetabular cup in 190 implantations via a direct anterior approach and a minimally invasive anterolateral approach in supine position performed by a single surgeon was analysed. In this cohort, a difference in planning adherence between the DAA and the MIS anterolateral approach in supine position could not be detected for the acetabular cup, the offset option of the stem and the stem size.

The results of the influence of surgical approaches on planning adherence, especially minimally invasive approaches, are mixed. Shemesh et al. [8] did not find a statistically significant difference in planning adherence within ± 1 size between the DAA and PA (85% vs. 77%; *p* = 0.71). Additionally, there was no significant difference in predicting the final stem’s neck angle or femoral offset. Rivera et al. [6] investigated the influence of surgical approach on planning adherence with Fitmore^®^ short stem system. A non-agreement for the femoral component was found in 42.37% in the PA and in 66.04% in the DAA without fluoroscopy. The frequency of stems at least two times smaller than templated was more than six times higher with the DAA compared to the PA (24.53% vs. 3.39%; *p* = 0.001). Similarly the planning adherence for Fitmore^®^ hip stem was shown for AL MIS approach [3]. The exact offset option as planned was implanted in 70.6% and the exact offset option and stem size as templated was only implanted in 21.6% [3]. The results in the present study shows similar results for Fitmore^®^ hip stem in the DAA and AL MIS group. The planning adherence for the offset option was comparable with 66.4% for the DAA in comparison to 55.6% in AL MIS without any statistical significance between both groups. Also, the planning adherence for stem size was without any statistical significance. However, the number of stems within ± 1 size and ± 2 sizes was higher in the DAA groups. Therefore, the DAA in combination with Fitmore^®^ hip stem shows less planning adherence for stem size. One of the major technical challenges in MIS approaches is femoral exposure and canal preparation, especially with the DAA [6–9, 13]. The results in the present study indicate, that the approach affects the planning adherence for stem size as the DAA shows a lower planning adherence compared to the AL MIS approach. However, the planning adherence for offset option is comparable for both MIS approaches, indicating a minor effect of the approach on the planning adherence for the offset option of Fitmore^®^ hip stem.

Apart from surgical approach the femoral component itself plays a role in planning adherence. Existing data concerning digital templating in total hip arthroplasty mainly focuses on straight stem systems. Holzer et al. [14] predicted the correct femoral component size for a straight stem system in 42% and within a range of ± 1 size for 87%. Jung et al. [5] compared planning accuracy between a straight stem and the Fitmore^®^ short curved stem design. The exact size of the femoral component was estimated correctly between 34.4% and 12.5% for the short stem by three surgeons with different experience levels compared to 42.9%, 39.3% and 28.6% in straight stem THA. Schmidutz et al.[4] compared the planning accuracy between short stem THA and straight stem THA without any significant difference. The planning adherence in the present study is comparable to other findings for Fitmore^®^ hip stem [3, 5, 6]. One reason for the lower planning adherence for the offset option could be the use of an a.-p. X-ray of the pelvis [3]. Merle et al. [15] demonstrated an increased risk for underestimation of femoral offset on AP radiographs of the pelvis compared with CT. In 13% the estimated femoral offset on AP radiographs was smaller than it was found with CT-based measurements. Additionally, Fitmore hip stem offers 4 different offset options with 14 sizes for each offset option. Therefore, a high number of possibilities are offered by this system, giving more possibilities of overruling the digital template. Therefore, we conclude, that the planning adherence for offset option in Fitmore hip stem is less influenced by the approach.

Patient specific factors showed a minor influence on planning adherence in the presented study for both approaches. Only the diagnosis of AVN or mild hip dysplasia was a significant risk factor for predicting the offset option of the femoral component. The effect on the stem size was not provable. The influence of BMI did not affect the predictability of any of the components in both groups. The effect of BMI on planning adherence is inconsistent in the literature. Comparable studies could not support an influence on planning adherence [3, 16], while other studies did show significant influence [14, 17]. Obesity could be a risk factor for mispositioning of the external calibration marker [17]. Boese et al. [18] considered the use of a radiopaque ball as calibration marker as a potential cause of error in digital templating. They demonstrated a statistically significant difference between the magnification factor based on internal magnification markers such as contralateral THA and external calibration They demonstrated a statistically significant difference between the magnification factor based on internal magnification markers such as contralateral THA and external calibration markers. In the presented study BMI was not detected as a risk factor for the planning adherence. Average BMI of the included patients was similar in both approaches without any statistical significance. Also, the distribution of patients divided in BMI-subgroups did not show any significant differences. Obesity might be an impairing factor because of reduced surgical exposure in minimally invasive approaches. However, a significant effect could not be detected in the presented study, given a similar number of severely obese patients with a BMI ≥ 35 kg/m^2^ without significant difference in testing. The overall number of severely obese patients is generally low. Therefore, a higher number of severely obese patients might be needed to give a clear answer on whether BMI influences the planning adherence.

An additional reason for the low planning accuracy regarding stem size could be a higher an acceptance of lower Canal Fill Indices. For Fitmore^®^ hip stem Canal Fill Indices different canal fill indices are described between 79.85 and 85.85% for DAA and 80.25–83.81% for AL MIS approach [7]. Comparable studies report higher Canal Fill Indices between 85.2 and 90.4% [19]. Therefore, a possible reason for lower planning accuracy could be accepting lower stem sizes than templated when rotational stability is intraoperatively achieved.

The planning adherence for the bi-hemispheric acetabular cup was similar between the DAA with 44.9% and the AL MIS with 41.7%. The number of cups within ± 1 size was higher in AL MIS approach. However, the number of cups within ± 2 sizes was slightly higher in the DAA. The planning adherence for the acetabular cup within ± 1 size is lower with 78% for the DAA compared to 87,5% in the AL MIS approach. The approach did not influence the planning adherence significantly in the presented study. Therefore, the results in the presented study indicate a lower accuracy for the acetabular cup in the DAA. However, results are not statistically significant in testing and logistic regression. These results for the acetabular cup are comparable to other studies [3, 5, 14]. Holzer et al. found a planning adherence of 75.5% within ± 1 size in lateral transgluteal approach [14]. The planning adherence for the Allofit^®^ cup in AL MIS approach is described with 74.8% within ± 1 size [3].

Limitations of this study are the retrospective design. The biggest limitation is the use of AP radiographs of the pelvis for digital templating as shown previously [3]. The underestimation of femoral offset was clearly shown by Merle et al. [15]. An improvement of planning accuracy could have been expected with the additional use of radiographs of the affected hip. Additionally, fluoroscopy was not routinely used in this case series. The use of fluoroscopy may be an effective tool for improving planning adherence by preventing intraoperative misconceptions. However, the benefit of fluoroscopy is controversial [10, 19, 20]. Especially in the DAA in combination with Fitmore hip stem, routinely use of fluoroscopy might be a feasible option for preventing undersizing of the femoral component due to the impaired femoral exposure. An additional limitation is the case number. Especially for the regression analysis a higher number of cases might have an influence on giving more exact results for the patient specific factors BMI and diagnosis. The number of patients with AVN or hip dysplasia is too low to give clear results for the stratification in regression analysis.

## Conclusion

Digital templating for THA with an uncemented short curved stem and a bi-hemispherical acetabular cup show comparable results between a direct anterior approach and a minimally invasive anterolateral approach in supine position. Surgeons should be aware of a low planning adherence for this type of short stem in minimally invasive approaches.

## Data Availability

Data and materials are available on request.
